# Supplementary material for: Stable flies are bona fide carriers of mastitis-associated bacteria
Source: mSphere. 2024 Jun 26;9(7):e00336-24. doi: 10.1128/msphere.00336-24 (PMC11288000; doi:10.1128/msphere.00336-24)
Supplement: File S5 — Figures S1-S16. [file msphere.00336-24-s0005.pdf]

Supplementary information for: Stable flies are bonafide carriers of mastitis-associated bacteria

Andrew J. Sommer[1,2], Julia E. Kettner[2], Kerri L. Coon[2]#

[1]Microbiology Doctoral Training Program, University of Wisconsin-Madison, Madison, WI 53706 USA

[2]Department of Bacteriology, University of Wisconsin-Madison, Madison, WI 53706 USA

#Address correspondence to Kerri L. Coon, kerri.coon@wisc.edu.

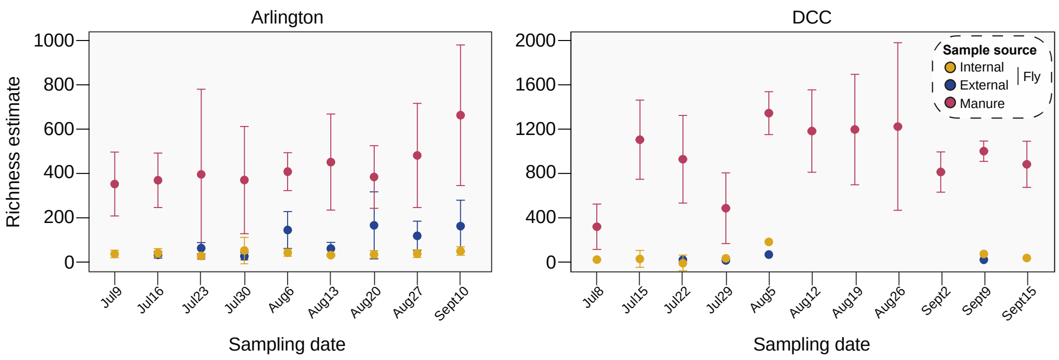

Fig. S1 Community-level ASV richness in Arlington- (*left*) and DCC-derived fly and manure samples (*right*), by sampling date (*x*-axis). Points are colored by sample source (internal fly samples, yellow; external fly samples, blue; manure samples, red). Bars represent 95% CI (± 1.96 * SE).

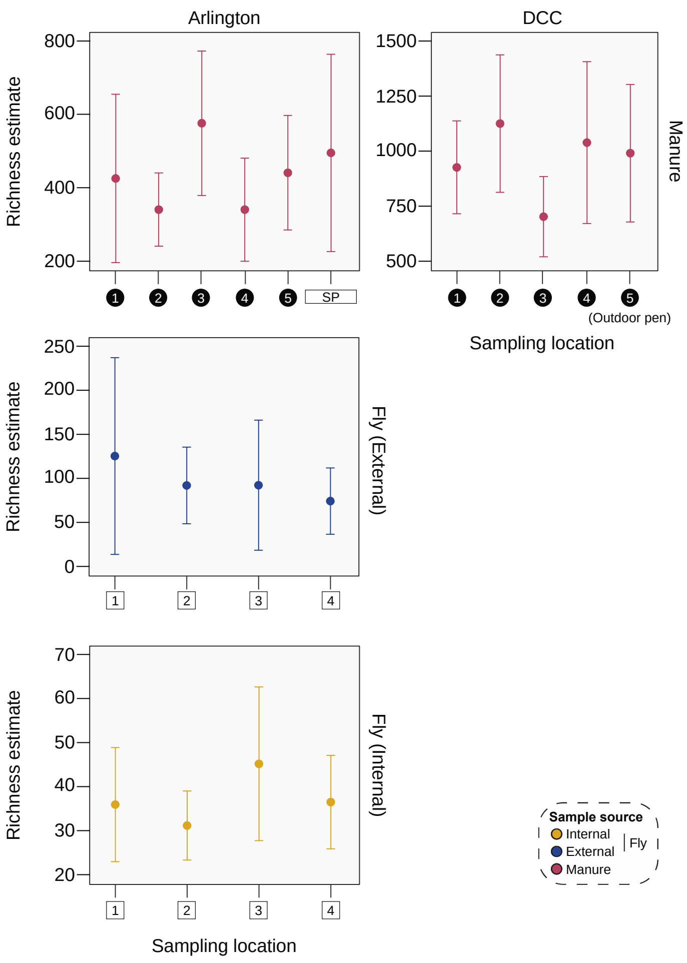

Fig. S2 Community-level ASV richness in Arlington- (*left*) and DCC-derived fly and manure samples (*right*), by sampling location (*x*-axis; see Figure 1 for more information). Points are colored by sample source (top panel: manure samples, red; middle panel: external fly samples, blue; bottom panel: internal fly samples, yellow). Bars represent 95% CI (± 1.96 * SE).

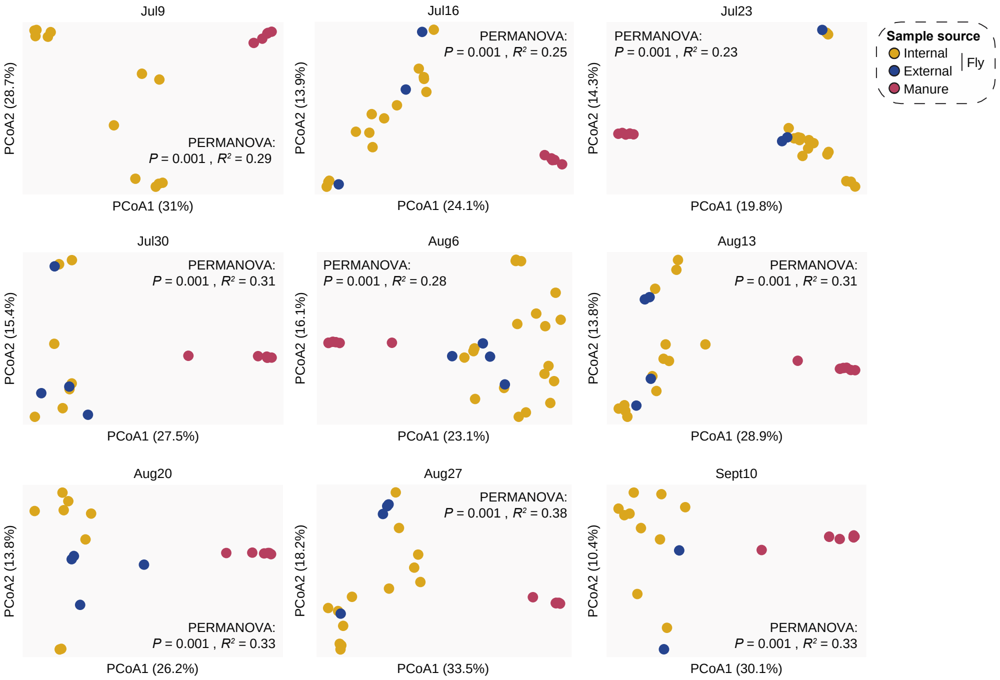

Fig. S3 PCoA of Bray-Curtis dissimilarities of community relative abundances, by sampling date. Points are colored by sample source (internal fly samples, yellow; external fly samples, blue; manure samples, red), with each point representing one Arlington-derived manure or fly sample community. PERMANOVA identified significant clustering by sample source for all sampling dates (P = 0.001, R^2 = 0.23-0.38).

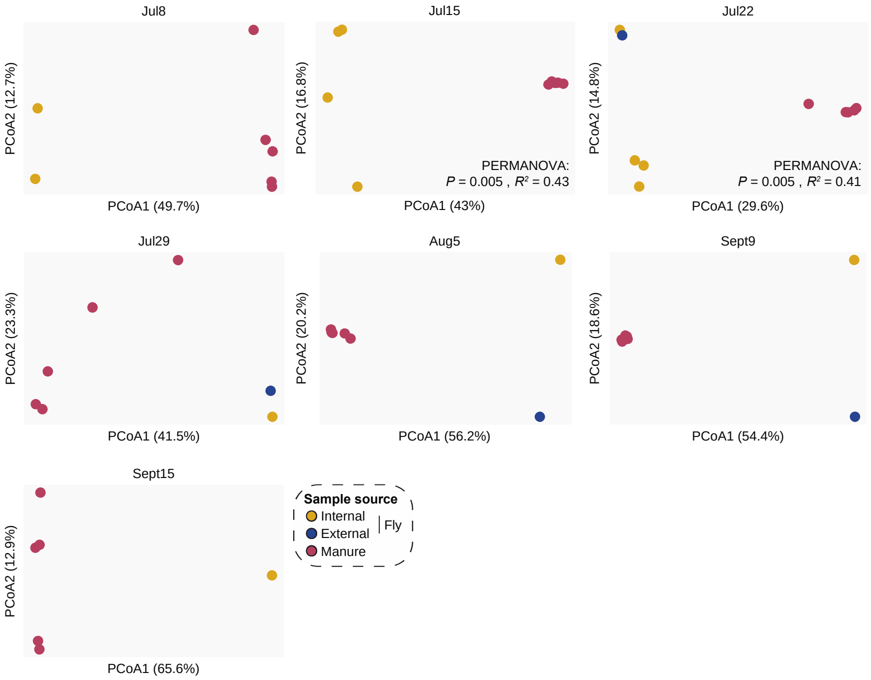

Fig. S4 PCoA of Bray-Curtis dissimilarities of community relative abundances, by sampling date. Points are colored by sample source (internal fly samples, yellow; external fly samples, blue; manure samples, red), with each point representing one DCC-derived manure or fly sample community. PERMANOVA identified significant clustering by sample source for both sampling dates for which there were enough samples to compare communities (Jul15: $P = 0.008$, $R^2 = 0.43$; Jul22: $P = 0.007$, $R^2 = 0.41$).

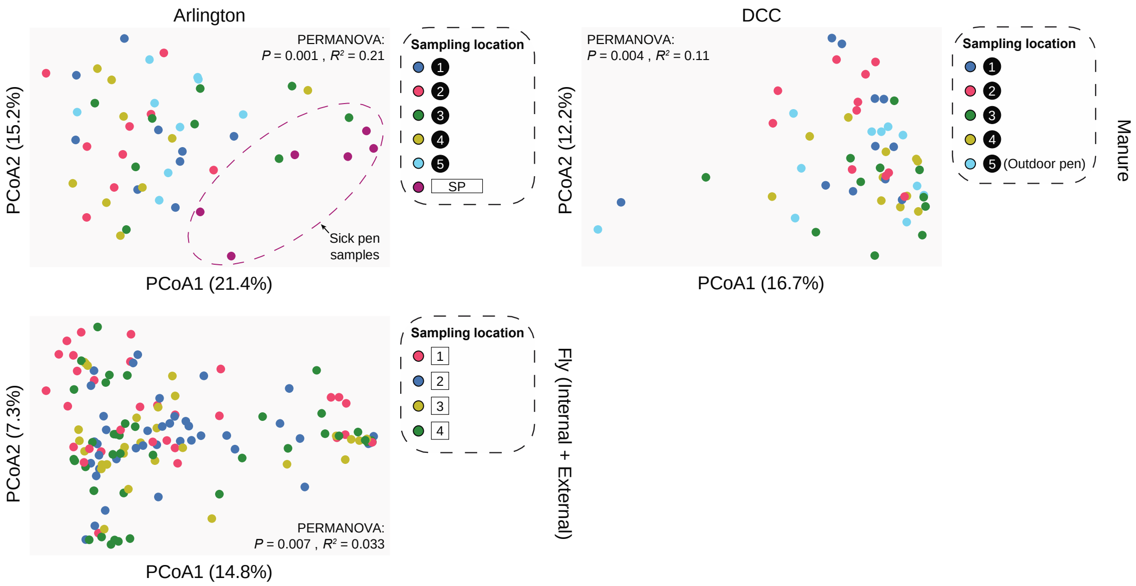

Fig. S5 PCoA of Bray-Curtis dissimilarities of community relative abundances, colored by sampling location (see Figure 1 for more information). Each point represents one Arlington- (*left*) or DCC-derived manure (*top*) or fly *(bottom*) sample community. PERMANOVA identified significant clustering by sampling location for both Arlington- (manure: P = 0.001, R^2 = 0.21; fly: P = 0.003, R^2 = 0.03) and DCC-derived samples (manure: P = 0.006, R^2 = 0.11). However, significant *post hoc* pairwise comparisons were only identified for Arlington- and DCC-derived manure samples, including those between samples collected from the Arlington sick pen and other sampling locations at the same facility ($P < 0.05$). No significant pairwise comparisons were identified for Arlington-derived fly samples ($P > 0.05$).

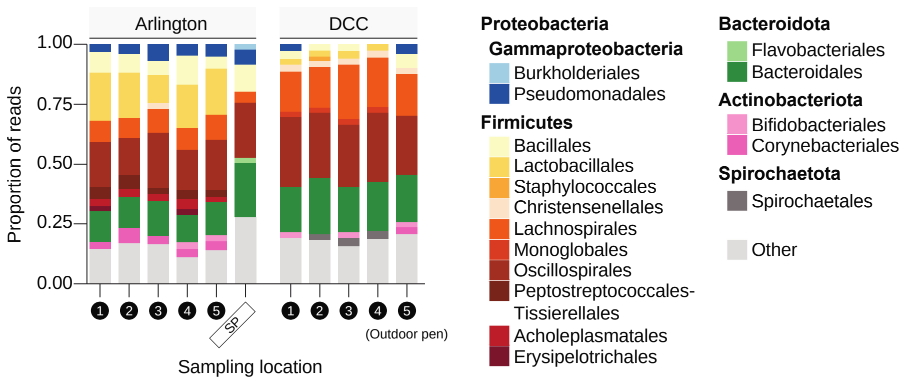

Fig. S6 Relative abundance of bacterial orders in Arlington- (*left*) and DCC-derived manure samples (*right*), by sampling location (*x*-axis; see Figure 1 for more information). Libraries derived from samples collected at a given sampling location were pooled across sampling dates for the bar graphs presented. Colored bars present the proportion of sequencing reads assigned to a given bacterial order. Low abundance orders (<2%) are represented by the “Other” category”.

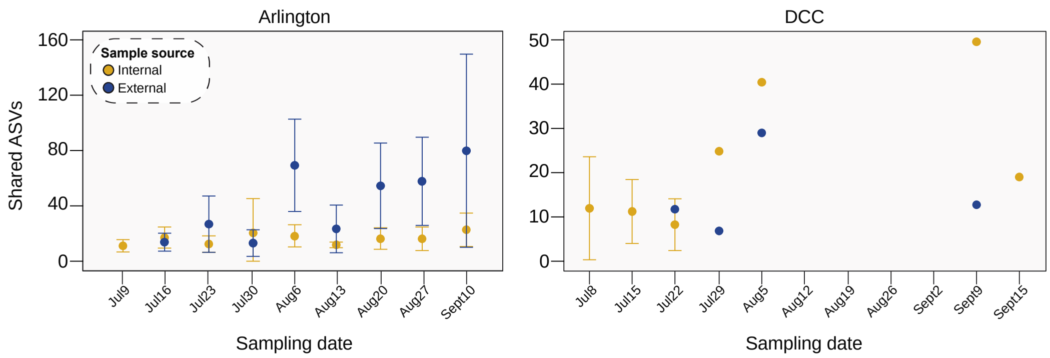

Fig. S7 Average number of ASVs shared between Arlington- (*left*) or DCC-derived fly (internal or external) and manure samples (*right*), by sampling date (*x*-axis). Points are colored by sample source (internal fly samples, yellow; external fly samples, blue). Bars represent 95% CI (± 1.96 * SE).

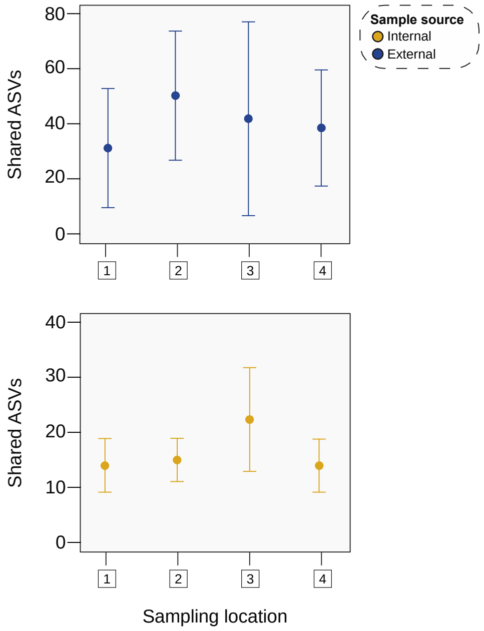

Fig. S8 Average number of ASVs shared between Arlington-derived fly (internal or external) and manure samples, by sampling location (*x*-axis; see Figure 1 for more information). Points are colored by sample source (top panel: external fly samples, blue; bottom panel: internal fly samples, yellow). Bars represent 95% CI (± 1.96 * SE).

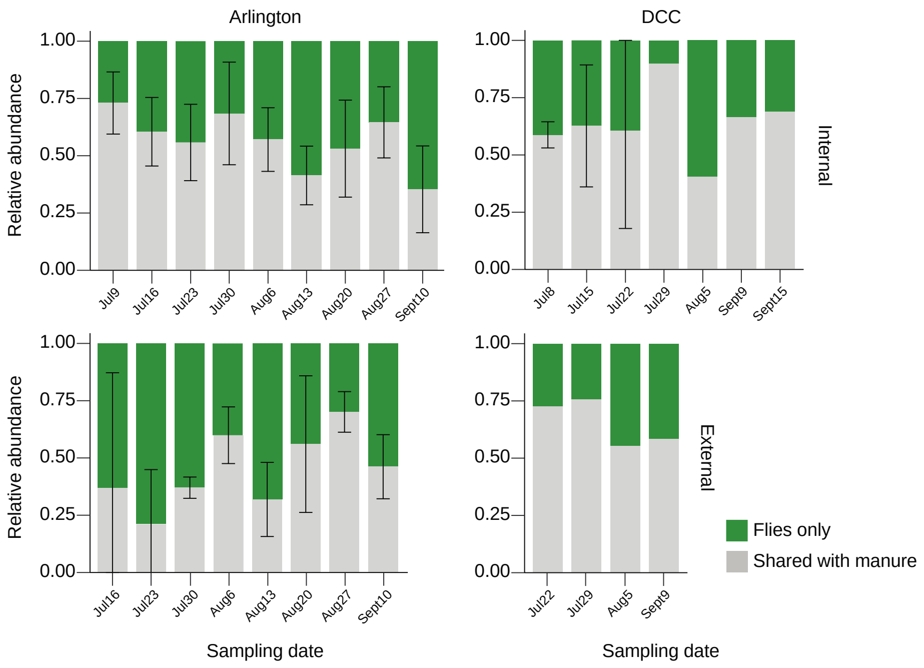

Fig. S9 Average relative abundance of fly-associated ASVs (internal or external) shared with manure collected from Arlington (*left*) or the DCC (*right*), by sampling date (*x*-axis). Bars represent 95% CI (± 1.96 * SE).

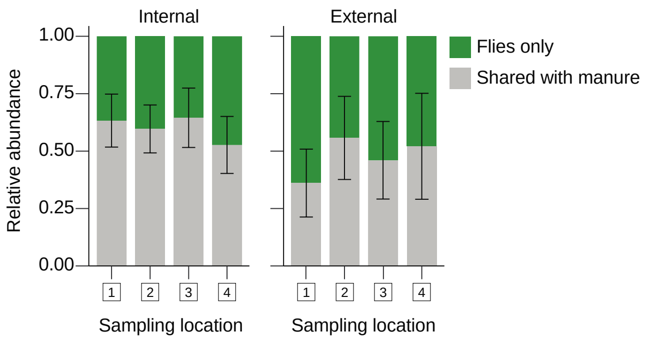

Fig. S10 Average relative abundance of fly-associated ASVs (internal or external) shared with manure collected from Arlington, by sampling location (*x*-axis; see Figure 1 for more information). Bars represent 95% CI (± 1.96 * SE).

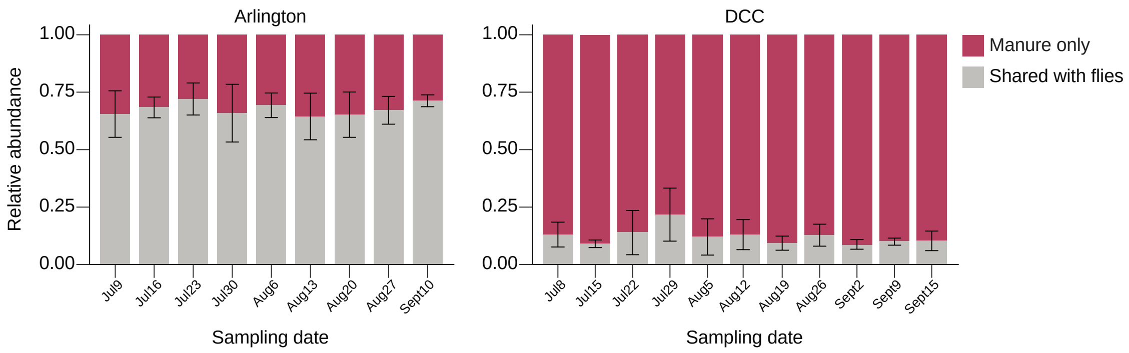

Fig. S11 Average relative abundance of manure-associated ASVs shared with flies collected from Arlington (*left*) or the DCC (*right*), by sampling date (*x*-axis). Bars represent 95% CI (± 1.96 * SE).

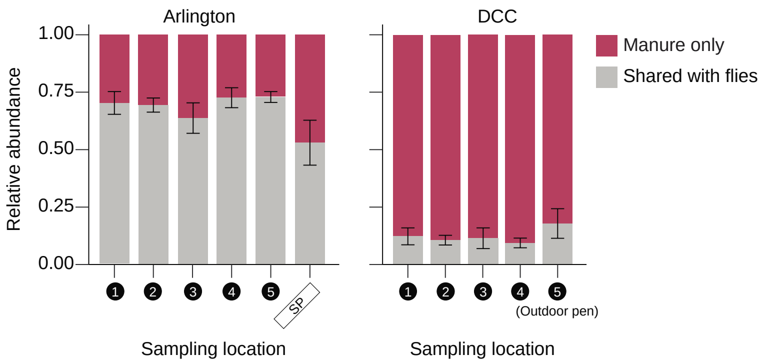

Fig. S12 Average relative abundance of manure-associated ASVs shared with flies collected from Arlington (*left*) or the DCC (*right*), by sampling location (*x*-axis; see Figure 1 for more information). Bars represent 95% CI (± 1.96 * SE).

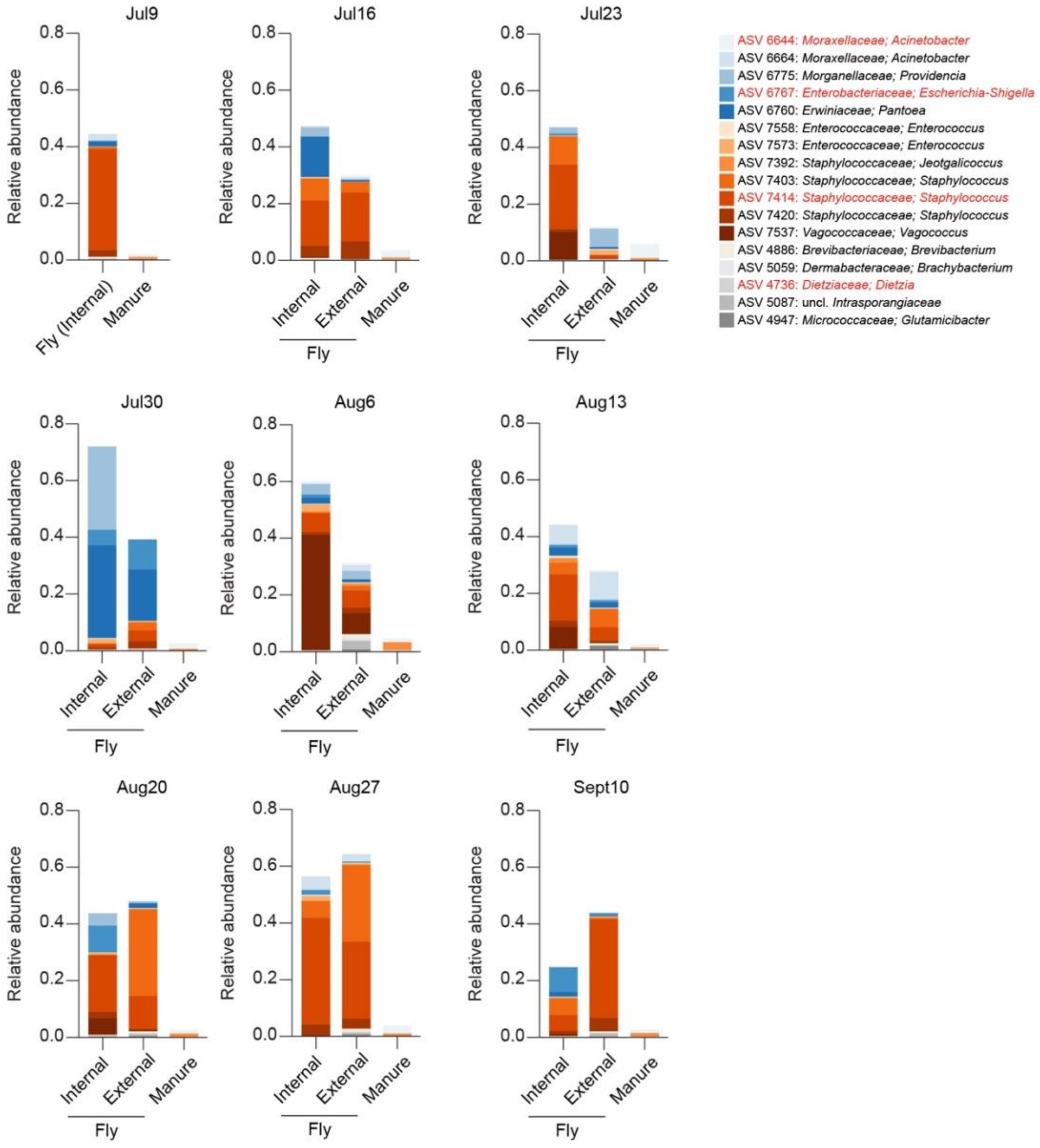

Fig. S13 Relative abundance of commonly shared ASVs in Arlington-derived fly and manure samples, by sampling date. Libraries derived from the same sample source on a given sampling date were pooled across sampling locations for the bar graphs presented. Colored bars present the proportion of sequencing reads assigned to a given ASV. ASV taxonomy is shown at the genus level. Taxa highlighted in red were identified as commonly shared ASVs in both facilities.

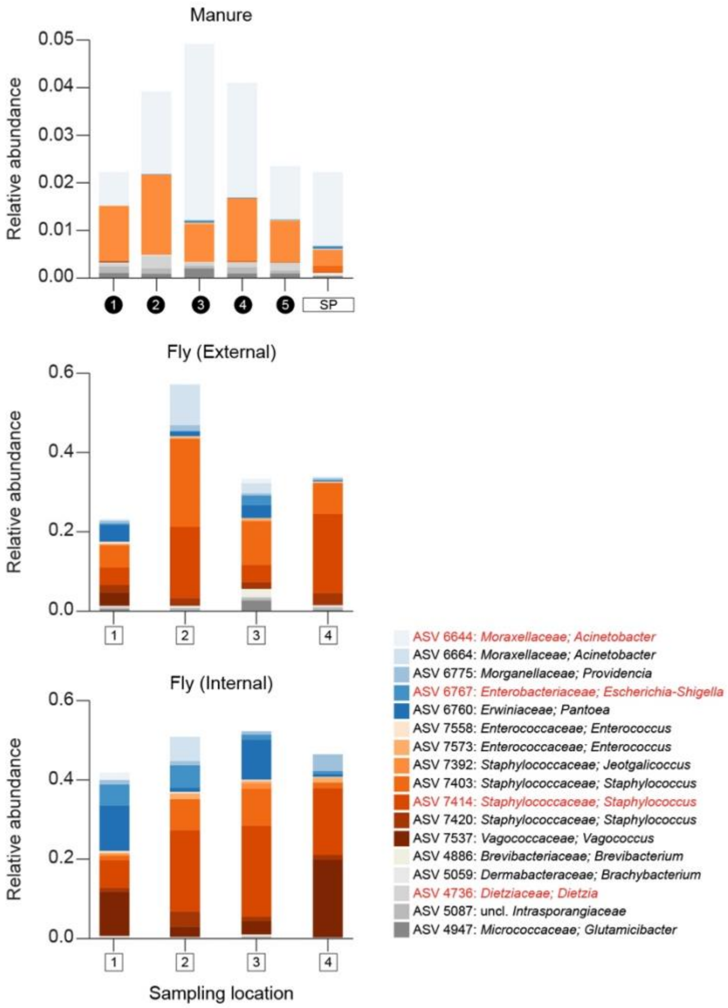

Fig. S14 Relative abundance of commonly shared ASVs in Arlington-derived fly and manure samples, by sampling location (*x*-axis; see Figure 1 for more information). Libraries derived from samples collected from the same sample source at a given sampling location were pooled across sampling dates for the bar graphs presented. Colored bars present the proportion of sequencing reads assigned to a given ASV. ASV taxonomy is shown at the genus level. Taxa highlighted in red were identified as commonly shared ASVs in both facilities.

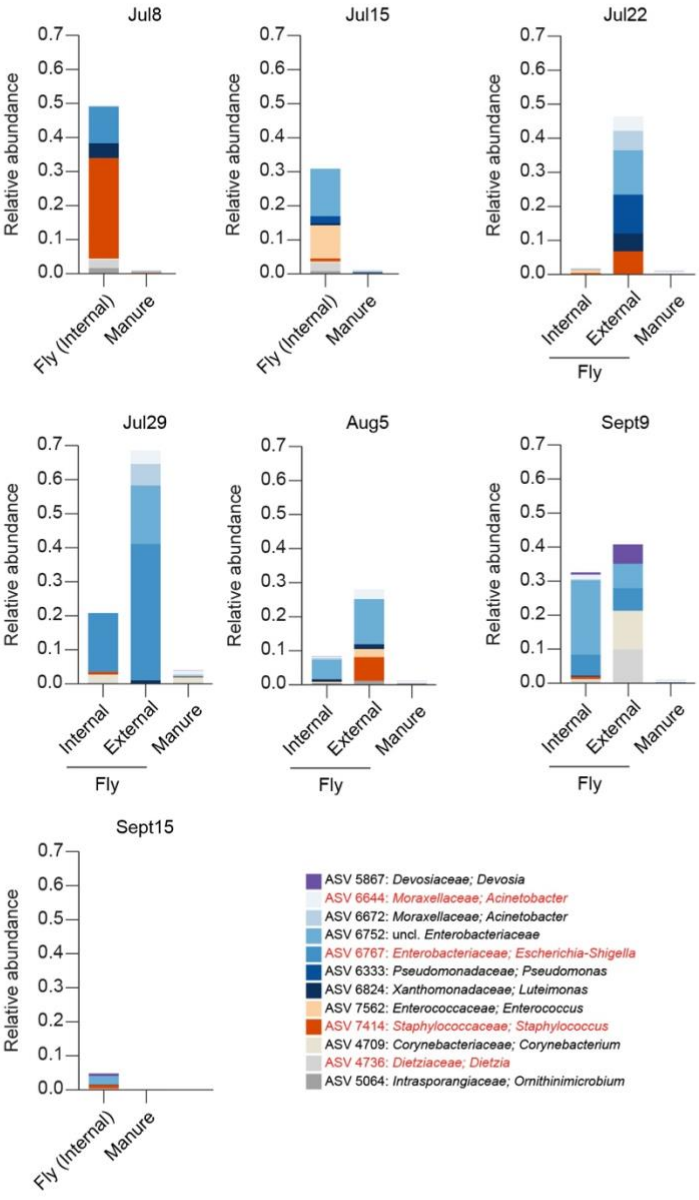

Fig. S15 Relative abundance of commonly shared ASVs in DCC-derived fly and manure samples, by sampling date. Libraries derived from the same sample source on a given sampling date were pooled across sampling locations for the bar graphs presented. Colored bars present the proportion of sequencing reads assigned to a given ASV. ASV taxonomy is shown at the genus level. Taxa highlighted in red were identified as commonly shared ASVs in both facilities.

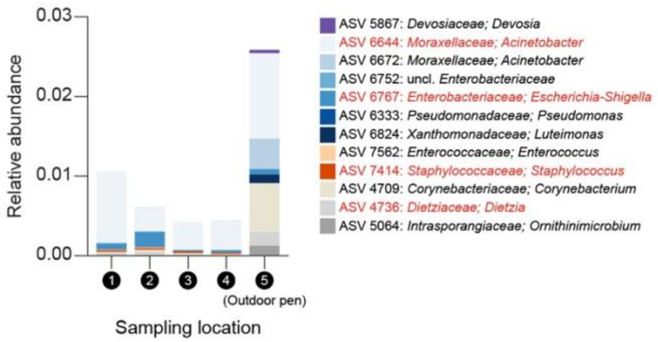

Fig. S16 Relative abundance of commonly shared ASVs in DCC-derived manure samples, by sampling location (*x*-axis; see Figure 1 for more information). Libraries derived from samples collected at a given sampling location were pooled across sampling dates for the bar graphs presented. Colored bars present the proportion of sequencing reads assigned to a given ASV. ASV taxonomy is shown at the genus level. Taxa highlighted in red were identified as commonly shared ASVs in both facilities.
